# Automated vision screening of children using a mobile graphic device

**DOI:** 10.1038/s41433-021-01862-x

**Published:** 2021-12-06

**Authors:** Steven A. Kane, Mark Gaspich, Julia Kane, Sarah A. Weitzman, Albert Hofeldt

**Affiliations:** 1 Vagelos College of Physicians and Surgeons, Columbia University, The Edward S. Harkness Eye Institute, 635 West 165th Street, Room 372, New York, USA; 2 University of Central Florida, Orlando, FL USA; 3 Syracuse University, Syracuse, NY USA; 4 New York Eye and Ear Infirmary of Mount Sinai, New York, NY USA

**Keywords:** Paediatrics, Vision disorders

## Abstract

**Background/Objective:**

Can measuring interocular brightness disparity, acuity, and colour vision classify children with amblyopia?

**Subjects/Methods:**

Two hundred eight subjects (3–14 years) were recruited for a prospective, observational protocol to measure interocular brightness disparity, uniocular acuities with and without a pinhole, and colour vision using an iPad. Subjects looked through polarizing filters and chose the brighter of two spaceships to measure interocular brightness disparity. The differential brightness of image pairs was varied through a staircase algorithm until equal brightness was perceived. Acuities and colour vision were tested with tumbling Es and AO-HRR colour plates, respectively. Unilateral amblyopia was later confirmed in two subjects.

**Results:**

Binocular brightness balance on the iPad detected amblyopes with 100% sensitivity and specificity. Using 20/30 as cutoff for normal acuity, 1 of the amblyopes was detected, and non-amblyopes were excluded by visual acuity pinhole testing. The mean difference between iPad and E-Chart visual acuities with pinhole was 0.02 logMAR with limits of agreement from −0.08 to +0.11 logMAR. iPad and printed plates Colour vision testing produced identical results. Testing times were brief and exit pleasure responses were positive. Mean and range testing times for Brightness Sense, Colour vision, and Visual Acuity were 32.7 s (range = 12–63 s), 52.8 min (range = 17–95 s), and 88.75 s (range = 41–188 s), respectively.

**Conclusions:**

Interocular brightness disparity, acuity, and colour vision can be measured in children as young as 3 years old solely through playing a game on a mobile device. Interocular brightness disparity is a sensitive and specific method to detect unilateral amblyopia.

## Introduction

Amblyopia, an often silent and elusive disease, remains the leading cause of permanent vision loss in children [1] despite more than a century of interest in vision screening [2]. Are the screening techniques at fault, are follow up and therapy at fault, or are not enough children being screened for amblyopia? Ideal vision screening would have low rates of false positive and false negative results, low expense, and readily availability. A technique having these qualities that could also be administered via telemedicine could improve vision screening in schools and paediatric offices and reach more children who are not being screened for amblyopia.

Relative brightness sense was found to agree closely with the degree of visual acuity impairment in adult subjects with a range of ophthalmic diseases, including amblyopia [3]. This study investigates the utility of vision screening with a mobile graphic device (iPad) to measure interocular brightness disparity, visual acuity, and colour vision for detecting amblyopia in paediatric subjects in a school setting.

## Subjects and methods

**Fig. 1:**
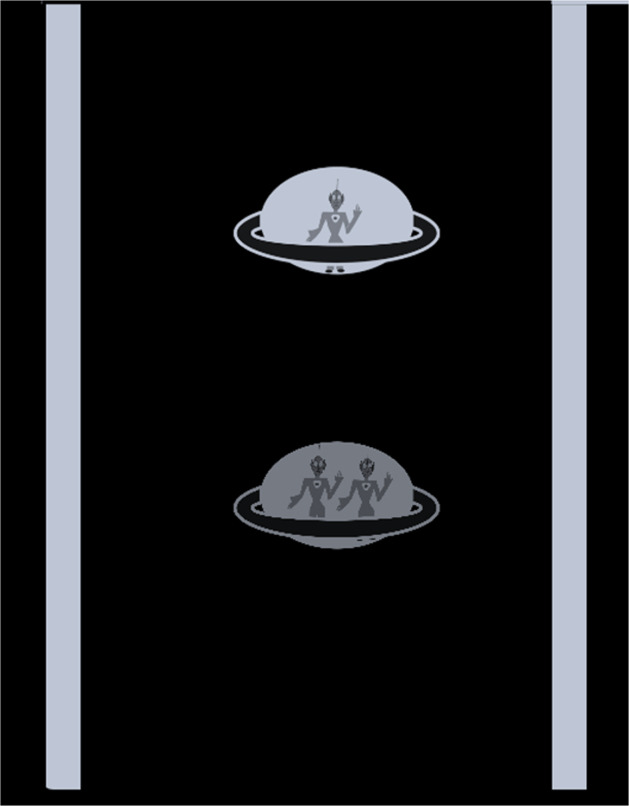
Screenshot of two spaceships presented on an iPad. After wearing polarizing glasses and looking at linear polarizing filters over two spaceships on an iPad, binocular image separation is created in order to measure brightness disparity.

This study utilized a prospective, observational protocol that followed all the tenets of the Declaration of Helsinki and was approved by the Columbia University Institutional Review Board (protocol AAAC0020). 208 children, 121 girls and 87 boys with ages from 3 to 14 years and mean of 7.8 years, were recruited as subjects and tested at one school, following informed consent. The protocol measures brightness disparity, visual acuity, and colour vision with self-tested algorithms running on an iPad with results stored on the device. The school was given iPads, filters, glasses, and pinholes (PHs) for the testing period, and the application needed is free for download on the iPads provided. The age distribution of these subjects was 15.4% (3–5 years of age), 46.6% (6–9 years of age), and 37.9% (10–14 years of age). Their ocular histories were not known beyond the use of spectacles until testing was completed. If a child wore glasses, the child was only tested while wearing those glasses. To measure brightness disparity, binocular image separation is created by wearing polarizing glasses combined with complementary linear polarizing filters positioned over two vertically aligned spaceships on an iPad screen (Fig. 1). Through this polarizing filter arrangement, the right eye views only the bottom spaceship, rivalrous with the black background viewed by the left eye, and the left eye views only the top spaceship, rivalrous with the black background viewed by the right eye. Top and bottom spaceships are presented with brightness differences ranging from 0.3 to 1.8 log units in increments of 0.3. In response to recorded instruction, the subject identifies and taps the brighter spaceship. The brightness difference of the spaceships and response times are recorded on the device. In response to the subject’s selection of the brighter spaceship, brightness differences of subsequent spaceship pairs are then sequenced within a stepwise, self-tested algorithm until the right-left brightness equality endpoint is crossed and re-crossed. For a normal score the students must achieve a net zero brightness imbalance in two of three games.

iPad visual acuity is based on matching tumbling Es calibrated from 20/400 to 20/20 for a testing distance of 40 cm. A tape measure attached to an iPad stand confirms the testing distance. Reversible spectacles that occlude one eye are worn. Testing begins by the trained examiner selecting a starting E size, typically 20/60. Most subjects began testing at the 20/60 level, but if the student failed to read the smaller letters at 20/60, the testing was then re-started at the largest level of 20/400. Two equally sized Es are presented. The student taps YES on the touchscreen when the orientations of the Es are identical and NO when the Es are mismatched. Three correct responses advance the protocol to the next lower line. An incorrect response provides a second chance during presentation of three E pairs of that letter size. Another incorrect response at that E size terminates the self-test. Testing then restarts by the same trained examiner selecting a larger starting E size. The smallest E size with 3 correct responses is recorded as the visual acuity for each eye. When visual acuity measures 20/30 or worse, testing is repeated through a plastic panoramic PH disc containing seven 6 mm opaque rings, each with a 1.0 mm central piercing and each ring margin separated by 1 mm of clear plastic. If the subject fails to match the 20/400 E, the acuity is recorded as less than 20/400. For comparison to distance acuity, the acuities of 63 subjects were also tested with tumbling Es (E-Chart) on a traditional eye chart at 20 feet.

Digital copies of the demonstration and test AO-HRR colour plates are presented on an iPad. Reversible spectacles that occlude one eye are worn. The subject is asked to touch a coloured shape or signify no coloured shape by touching a no colour circle below. If the demonstration plates are correctly identified, the subject qualifies to proceed. The test colour images are then similarly presented in a pseudo-random order and then repeated for the other eye.

Subjects with abnormal results were referred for complete ophthalmological examination if they were not already under care.

## Results

Of the 208 subjects were recruited for testing, 204 subjects were able to complete the protocol to measure interocular brightness disparity, acuities, and colour vision. Except for one amblyope, the visual acuities of the remaining 203 subjects were 20/30 or better in each eye either unassisted, with corrective spectacles, or with the aid of the PH. Four subjects were excluded from the protocol due to either not understanding the visual acuity tests (two students) or omission of PH acuity testing (two subjects). The only recruited subject who was unable to successfully play the brightness disparity game was a young child who was also unable to perform the acuity and colour vision tests. However, it has been demonstrated previously [4] that screening for amblyopia around the age of 5 rather than earlier may be the best scenario.

**Fig. 2:**
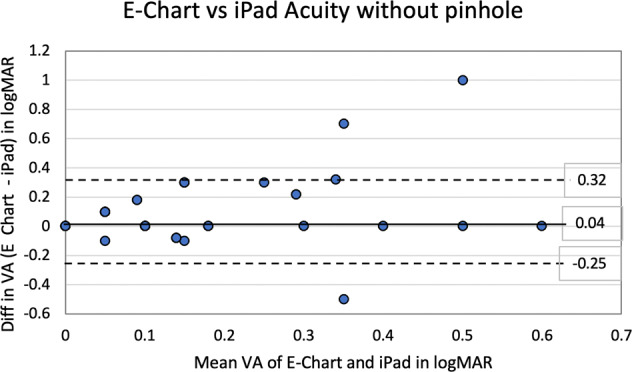
Analysis of iPad Acuity without pinhole. Bland–Altman plot of agreement between E-Chart and iPad visual acuities without pinhole for 63 subjects, 126 eyes.

**Fig. 3:**
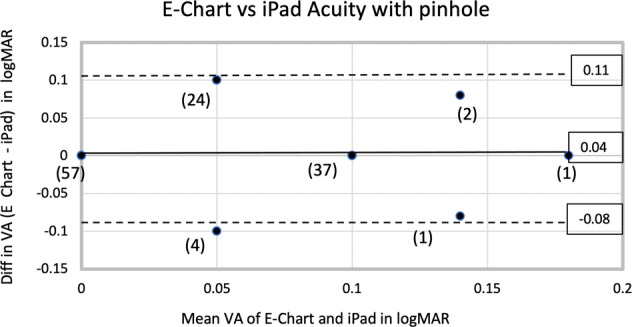
Analysis of iPad Acuity with pinhole. Bland–Altman plot of agreement between E-Chart and iPad visual acuities with pinhole for 63 subjects, 126 eyes, frequency (*n*).

For statistical analysis, acuities were converted to logMAR notation. For the 63 students (126 eyes) tested with both E-Chart and the iPad acuity without the PH, the mean logMAR acuities for E-Chart was 0.11 (standard deviation SD = 0.16) and for iPad acuity was 0.07 (SD = 0.12). With the PH, the mean logMAR acuity for E-Chart was 0.05 (SD = 0.05) and for iPad acuity was 0.04 (SD = 0.05). The improvement in mean visual acuity with the addition of the PH was 55% (0.11 vs 0.05) for E-Chart and 42% (0.07 vs 0.04) for iPad acuity while SD improved 67% (0.16 vs .05) for E-Chart and 58% (0.12 vs 0.05) for iPad acuity. The limit of agreement between logMAR iPad acuity and E-Chart was analyzed by the method of Bland and Altman [3], where 95% of differences will lie between plus and minus 2 SD of the mean difference (d) between the tests. Without the PH (Fig. 2), for E-Chart minus iPad acuity, *d* = 0.04 logMAR (SD = 0.14, *d* − 2SD = −0.25 and *d* + 2SD = 0.32). With the PH (Fig. 3), for E-Chart minus iPad acuity, *d* = 0.02 logMAR (SD = 0.05, d − 2s = −0.08 and *d* + 2s = 0.11).

Of the 204 subjects tested for brightness disparity, 2 had interocular brightness imbalance and 202 did not have brightness imbalance. When their ophthalmic status was unmasked, the first of these two subjects was known to have amblyopia and was under treatment, with a pre-treatment acuity of 20/60. This child being treated for amblyopia OS had acuities measuring 20/20 OD and 20/25 OS at the time of testing. Left brightness disparity thrice measured 0.3 log unit. Colour vision was normal in each eye. The second of these two subjects was not under ophthalmic care and was referred for complete ophthalmic examination. This child was confirmed to have previously undetected left amblyopia with acuities of 20/20 OD and 20/40 OS and no visual acuity improvement with PH. The subjects were tested with no prior knowledge of their visual status, which is the condition that is typical of school and paediatric screenings. After identifying abnormalities, the subjects were asked about any prior ophthalmic care that they had received or previous diagnoses. Left brightness disparity twice measured 0.6 log unit and once measured 0.3 log unit. Colour vision testing suggested a blue-yellow defect OS and normal colour vision OD. A third subject who had been successfully treated for amblyopia OS had acuities measuring 20/20 in each eye. Brightness disparity testing found alternating ocular preference with endpoints of 0.3 OS, 0.3 OD, and 0.0 log units. Colour vision was normal in each eye.

Of the 204 subjects tested for colour vision, 198 students tested normal, and 6 subjects displayed a colour vision defect using the AO-HRR colour plates in an iPad. Five with a defect were bilaterally identical, classified as hereditary, with one female (0.83% of the females) and 4 males (4.5% of the males). The remaining subject had a monocular colour vision defect in the amblyopic eye (the child with amblyopia described above). Two subjects (1%) were later confirmed to have unilateral amblyopia.

The target subjects for this study were those 8 years old and younger. For brightness disparity, testing time was measured in 62 subjects, including 32 females and 30 males, who were a mean age of 5.9 years old with an age range from 3 to 8 years old. Times ranged from 12 to 63 s with the mean time of 32.7 s and standard deviation of 9.8 s. For iPad acuity, testing time was measured in 36 subjects, including 20 females and 16 males, who were a mean age of 5.25 years old with an age range from 3 to 8 years old. Times ranged from 41 to 188 s with mean of 89 s with standard deviation of 35.6 s. Most subjects began testing at the 20/60 level (smaller letters on the eye chart), and if the student failed to read the smaller letters, the testing was re-started at the 20/400 level (largest letters on the eye chart); this difference was not factored into the recording time. For colour vision testing, testing time was measured in 38 subjects, including 20 females and 18 males, who were a mean age of 5.6 years old with an age range from 3 to 7 years old. Times ranged from 17 to 95 s with mean of 52.8 s and standard deviation of 25.4 s.

Exit pleasure polls on a scale from 1 (boring) to 10 (fun) were taken in 60 children. The average pleasure scores were 9.7 and significant with *p* = 0.05 for brightness disparity testing, 9.0 and significant with *p* = 0.0001 for iPad acuity, and 9.6 and not significant with *p* = 0.2 for colour vision testing. The younger subjects significantly reported higher pleasure scores than the subjects between 8 and 13 years of age.

## Discussion

The prevalence of amblyopia in this cohort is approximately 1%, within the reported prevalence of amblyopia worldwide between 1% and 4% [5]. Prevalence in our cohort toward the lower end of this range may reflect our recruitment process. We hypothesize that many students at this school already receive private ophthalmic care. Parents of some children with known amblyopia and other ocular conditions may have chosen to not respond to our invitation to participate in this study, possibly decreasing our measured prevalence of amblyopia.

The standard method for detecting amblyopia remains complete ophthalmic examination and measurement of best-corrected acuity [6]. In primary care and school settings, commercially available instrument-based screening devices are common [6–10]. These devices are mostly designed to detect risk factors for amblyopia such as refractive error, strabismus, anisocoria, and media opacities rather than relative decreased acuity or amblyopia. These risk factors occur in 21% [11] whereas amblyopia affects only 2–3% [1] of the population in the United States, a disparity that may explain the inverse relationship between sensitivity and specificity of these devices according to the referral criteria chosen by the manufacturer or operator [12]. Two more recently introduced devices, the Paediatric Vision Scanner [13] and Diopsys [14] objectively measure retinal birefringence and visually evoked potentials, respectively, to detect asymmetry between eyes and identify unilateral amblyopia. These devices are expensive, are not widely available in schools and paediatric offices, and are not readily applicable to telemedicine.

Why interocular brightness sense is useful to detect amblyopia is unknown. Many authors have found brightness sense useful for studying optic nerve disease. None to our knowledge state that normal brightness balance excludes disease. Inducing interocular brightness imbalance was found to severely impair hitting by major league baseball players [15], suggesting that brightness sense influences motion stereopsis and may be evolutionarily old and conserved. A study of colour rivalry suppression in patients with ocular disease and amblyopia suggested that brightness disparity might also accompany unilateral amblyopia [16]. Our study supports this hypothesis that measurement of interocular brightness sense while playing a game on a readily available mobile graphic device, an iPad, may be a sensitive and specific method to detect unilateral amblyopia. Specialized, expensive equipment is not needed for this testing, making this methodology potentially attractive for online vision screening and for telemedicine. The applications are available for free download, and disposable filters, glasses, and PH needed for testing is currently mailed upon request. The filters easily slip over an iPad or iPhone. This at-home testing requires an adult to assist the child in setting up and beginning the test, but once it is initiated, children find it incredibly easy to complete.

Acuity testing with iPad using tumbling Es was equivalent to distance testing in our cohort and compares favourably with other methods of acuity measurement. Without a PH, amblyopes are not segregated from those with only refractive error. In screening children having unknown refractive errors for amblyopia, we found that adding a panoramic PH improves acuities for iPad acuity and E-Chart to a level capable of excluding amblyopia with either eye chart. Applying Bland and Altman statistics [3], E-Chart verses iPad acuity with PH (*d* = 0.02, *d* + 2SD = 0.11, *d* − 2SD = −0.08) showed closer agreement than when other charts [17] were compared to E-Chart by this method: E-Chart vs HOTV (*d* = 0.17, *d* + 2s = 0.37, *d* − 2s = −0.03) and E-Chart verses Lea symbols (*d* = 0.15, *d* + 2s = 0.36, *d* − 2s = −0.07). Our limits of agreement (0.11 and −0.08) suggest that E-Chart and iPad acuity with panoramic PH can be used interchangeably. Combining measures of interocular brightness disparity and acuity with PH permits identification of unilateral amblyopia by two methods using one device.

The incidence of bilateral amblyopia has been estimated to be 0.5% and the interocular acuity difference can be very small, less than 1 lines of letters in 50% of the patients [18]. In our study, brightness disparity testing detected the amblyope with one line of difference in visual acuity, however, more studies are needed to determine the sensitivity for detecting a minimum interocular vision difference by this method. Until that sensitivity is known, both brightness disparity and acuity testing should be used for detecting amblyopia.

Customary visual acuity testing does not separate those with refractive errors from those with amblyopia. However, individuals referred for ophthalmic care can have amblyopia, refractive errors, or both. Brightness disparity testing is fast, fun and appears to be sensitive for identifying those with amblyopia. Those few detected with amblyopia, which is about 1–4% of those screened, would be labelled for urgent treatment and then closely followed. The worldwide problem is not the detection of refractive errors but rather the detection of amblyopia, which is the number one cause of permanent blindness in children.

Video games and smartphones and tablets are ubiquitous across many societies and popular with children. This study found measures of interocular brightness disparity and visual acuity using tumbling Es useful to detect amblyopia in young children. The determination of interocular brightness disparity required only an average of ½ minute testing time per eye, was easy in that only 1 young subject of the 208 subjects was unable to play the “game,” and was fun, with a mean exit pleasure score of 9.7/10. The rationale that earlier screening for amblyopia leads to better outcomes is being questioned [4], as is the value of current vision screening in children [19]. Outcomes were similar when treatment was immediately initiated or delayed [20], so screening when children are 3 years and able to play a video game remains a reasonable approach to lessening the societal burden of visual loss due to amblyopia.

Determination of brightness disparity with a graphic mobile device as demonstrated in this study is fun and easy for children and is highly sensitive and specific for detecting unilateral amblyopia. Acuity testing with spectacles or PH on the same device can support the results of brightness disparity measurement and help detect bilateral amblyopia. Online vision screening and telemedicine that directly measure amblyopia rather than assess risk factors may eventually displace amblyopia as number one cause of permanent vision loss in children. The screening of children in different schools and paediatric practices comparing this methodology with existing commercial devices is planned.

## Summary

### What was known before

- Amblyopia detection currently emphasizes detection of risk factors or the use of expensive devices that are not readily available Detection of amblyopia by its risk factors overestimates referrals and can fail to detect amblyopia.

### What this study adds

- Amblyopia can be detected by brightness rivalry. This technique is highly sensitive and specific. This method is inexpensive and utilizes readily available devices.
